# A low-loss and compact single-layer butler matrix for a 5G base station antenna

**DOI:** 10.1371/journal.pone.0226499

**Published:** 2019-12-16

**Authors:** Intan Izafina Idrus, Tarik Abdul Latef, Narendra Kumar Aridas, Mohamad Sofian Abu Talip, Yoshihide Yamada, Tharek Abd Rahman, Ismahayati Adam, Mohd Najib Mohd Yasin

**Affiliations:** 1 Department of Electrical Engineering, Faculty of Engineering, University of Malaya, Kuala Lumpur, Malaysia; 2 Department of Electronic Systems Engineering, Malaysia-Japan International Institute of Technology, Universiti Teknologi Malaysia, Kuala Lumpur, Malaysia; 3 Wireless Communication Centre, Universiti Teknologi Malaysia, Johor Bharu, Johor, Malaysia; 4 School of Computer and Communication Engineering, Universiti Malaysia Perlis, Arau, Perlis, Malaysia; 5 School of Microelectronic Engineering, Universiti Malaysia Perlis, Arau, Perlis, Malaysia; Information Technology University, PAKISTAN

## Abstract

Researchers are increasingly showing interest in the application of a Butler matrix for fifth-generation (5G) base station antennas. However, the design of the Butler matrix is challenging at millimeter wave because of the very small wavelength. The literature has reported issues of high insertion losses and incorrect output phases at the output ports of the Butler matrix, which affects the radiation characteristics. To overcome these issues, the circuit elements of the Butler matrix such as the crossover, the quadrature hybrid and the phase shifter must be designed using highly accurate dimensions. This paper presents a low-loss and compact single-layer 8 × 8 Butler matrix operating at 28 GHz. The optimum design of each circuit element is also demonstrated in detail. The designed Butler matrix was fabricated to validate the simulated results. The measured results showed return losses of less than −10 dB at 28 GHz. The proposed Butler matrix achieved a low insertion loss and a low phase error of ± 2 dB and ± 10°, respectively. In sum, this work obtained a good agreement between the simulated and measured results.

## Introduction

Research on the fifth-generation (5G) mobile communication systems has rapidly accelerated to meet the standardization of the International Telecommunications Union Radiocommunication by 2020 and its requirement for higher data rates and data traffic [1–4]. The features of 5G mobile communication systems include the utilization of millimeter waves, the deployment of small cells with a radius size of 200 m and the use of a multibeam base station antenna for multiple-input multiple-output schemes [2–7].

The multibeam array antenna is the most promising candidate for the 5G base station, as it reduces the co-channel interference by controlling the beam directions and improving network coverage [5, 8]. To achieve the multibeam characteristic, beamforming circuits such as the Butler matrix, the Rotman lens and the Blass matrix can be employed in the base station antenna [9–11]. The Butler matrix has received much attention due to its simple design, low power dissipation and cost-effectiveness for large-scale production [12]. The structure of this circuit is compact and therefore it can be mounted easily at the base station. The Butler matrix consists of three circuit elements, namely a crossover, a quadrature hybrid and a phase shifter. The performance of the Butler matrix depends on the performance of each circuit element. The main drawback of designing a Butler matrix is its high insertion losses, especially at millimeter wave for higher-order Butler matrices; thus affecting the overall performance of the circuit. Therefore, it is essential to use highly accurate circuit element dimensions to ensure the Butler matrix performs excellently.

Previous studies focused on the improvement of circuit performance and the size reduction of the Butler matrix at frequencies below 10 GHz by eliminating some circuit elements [13–15]. Babale, Rahim, Barro, Himdi and Khalily [13] developed a 4 × 4 Butler matrix using only modified quadrature hybrids without a phase shifter and a crossover to produce a phase difference of 45° at 6 GHz. The designed Butler matrix exhibited an insertion loss and a phase error of 3 dB and 3°, respectively. Moreover, Ben Kilani, Nedil, Kandil and Denidni [14] designed a 4 × 4 Butler matrix based on conductor-backed coplanar waveguide technology using two directional elliptic couplers, two directional slot-coupled couplers and two phase shifters to avoid the use of a crossover at 5.8 GHz. The proposed Butler matrix showed an insertion loss and a phase error of 1.5 dB and 10°, respectively. Tian, Yang and Wu [15] designed a 4 × 4 Butler matrix using hybrid couplers with phase differences of −45° and −90° along with a crossover at 6 GHz. The designed Butler matrix achieved a low insertion loss and a low phase error of 1 dB and 1°, respectively. However, the proposed hybrid couplers could not be characterized using the closed-form transmission line theory [13]. Although these research works successfully reduced the number of circuit elements, these techniques are only valid for a 4 × 4 Butler matrix and not applicable for higher-order Butler matrices, which have a more complex structural design.

Several designs of the Butler matrices have been reported at millimeter wave frequencies [16–18]. A Butler matrix operating at higher frequencies has a reduced physical size but an increased propagation loss, as the wavelength is smaller compared to lower frequencies. Trinh-Van, Lee, Yang, Lee and Hwang [16] presented a 4 × 4 Butler matrix consisting of four quadrature hybrids, two crossovers and two phase shifters at 27.925 GHz. The proposed Butler matrix obtained an average insertion loss and a phase error of 2 dB and 10°, respectively. Yang, Ban, Kang, Sim and Wu [17] reported a 4 × 4 Butler matrix based on a substrate integrated waveguide at 30 GHz. The Butler matrix designed in the study was composed of four quadrature hybrids, two crossovers and two pairs of phase shifters. The crossover was realized by cascading two quadrature hybrids. In addition, the phase compensation technique was employed to design 0° and 135° phase shifters. The proposed Butler matrix produced the simulated results of an insertion loss and a phase error of 1 dB and 5°, respectively. However, the authors did not report the measured results of an insertion loss and a phase error. Chen and Chu [18] designed a 4 × 4 Butler matrix based on a substrate integrated waveguide at 60 GHz. The Butler matrix designed in their study was formed using four quadrature hybrids, two crossovers and four phase shifters. The quadrature hybrids and crossovers were designed using short-slot couplers. The proposed Butler matrix achieved an insertion loss and a phase error of 2.5 dB and 12°, respectively. These research works were limited to a 4 × 4 Butler matrix and therefore not suitable for base station applications, as higher-order Butler matrices are required to feed the antennas at a base station.

Only a few research studies have reportedly developed 8 × 8 Butler matrices, as the structure of these Butler matrices are more complex and difficult to design. The main issues of designing an 8 × 8 Butler matrix are high insertion loss and high phase error, as it has more circuit elements compared to a 4 × 4 Butler matrix. Moubadir, Bayjja, Touhami, Aghoutane and Tazon [19] proposed an 8 × 8 Butler matrix based on a single-layer structure at 2.4 GHz for wireless local area network applications using electromagnetic simulation software. The Butler matrix consisted of twelve quadrature hybrids, four crossovers, four phase shifters with a −22° phase difference, two phase shifters with a −67.5° phase difference and two phase shifters with a −45° phase difference. However, the proposed Butler matrix was not validated via measurement. In addition, the circuit performance of the Butler matrix such as scattering parameters was not discussed. Zhai, Fang, Ding and He [20] designed an 8 × 8 Butler matrix at 4.3 GHz based on a dual-layer microstrip configuration using six quadrature hybrids and four phase shifters placed at the top and bottom layers, respectively. These layers were connected via through-holes. The Butler matrix yielded an insertion loss and a phase error of 2.5 dB and ± 15°, respectively. Moreover, Zhong, Ban, Lian, Yang, Guo and Yu [21] developed an 8 × 8 Butler matrix based on a dual-layer substrate integrated waveguide at 28 GHz to 31 GHz. The proposed Butler matrix consisted of an additional four quadrature hybrids as compared to the conventional configuration. The simulated results showed an insertion loss and a phase error of 2 dB and 15°, respectively. However, the study did not clarify the overall losses of the fabricated Butler matrix. The air gap between substrate layers can cause additional losses and phase errors, further degrading the performance of the Butler matrix. Moreover, the use of through-holes in multilayer technology increases fabrication difficulties. Based on the literature, these issues could be overcome using a single-layer microstrip structure that is cost-effective and simple in design.

The main challenge of this work was to develop an accurate circuit design for a Butler matrix with a low insertion loss and consistent transmission amplitude and phase difference between the output ports of the circuit. This paper presents the design methodology to achieve the desired electrical performance of a single-layer 8 × 8 Butler matrix at 28 GHz. The structure of the proposed Butler matrix is compact and therefore it is suitable for a practical space-constrained base station. This circuit was designed using three-dimensional electromagnetic simulation software. The designed Butler matrix was fabricated via a photo etching process. The measured results of the transmission amplitude distributions and the phase characteristics at the output ports of the Butler matrix were compared with the simulation results.

## Design of the Butler Matrix

**Fig 1:**
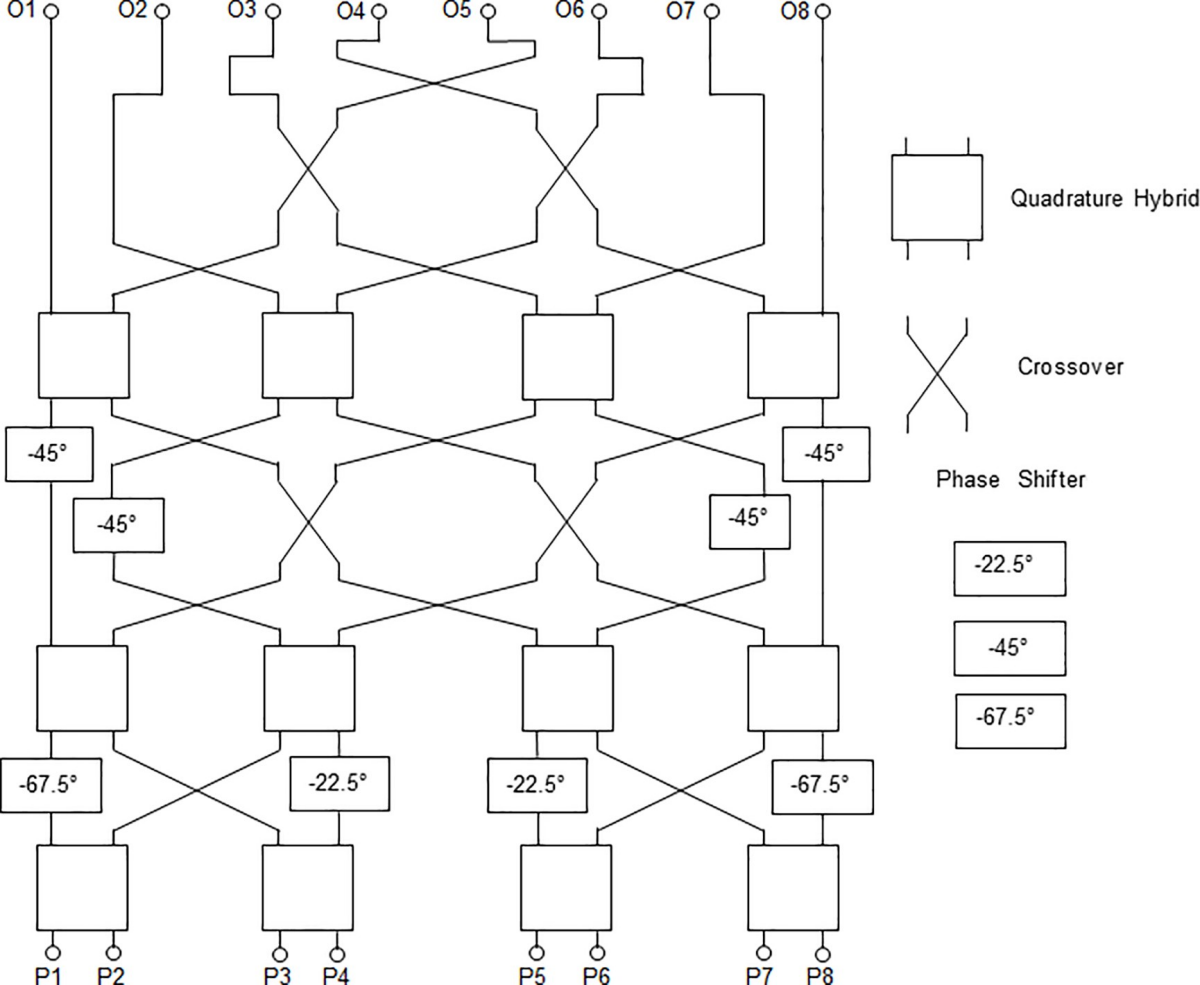
The structure of the 8 × 8 Butler matrix.

The Butler matrix is a passive feeding network of *N* inputs (Pi) and *N* outputs (Oi), where *N* is the power of 2 (*N* = 2^*n*^) [9]. It has equal amplitudes at the output ports when the input port is excited. The phase differences between the output ports for the *p*th beam angles are given by Eq (1) [22]:

$$\phi_{p}=\pm \frac{2p-1}{N}\times 180{}^{\circ}$$ (1)

where *N* = 8, *n* = 3 and *p* = 1, 2, …, (*n +* 1). Table 1 shows the phase differences between the output ports of the Butler matrix corresponding to Eq (1). The systematic design procedure of the Butler matrix can be referred to Moody [22]. The structure of the 8 × 8 Butler matrix is illustrated in Fig 1.

**Table 1:** The phase differences between the output ports of the Butler matrix.

*p*	*ϕ*_*p*_
1	± 22.5°
2	± 67.5°
3	± 112.5°
4	± 157.5°

**Fig 2:**
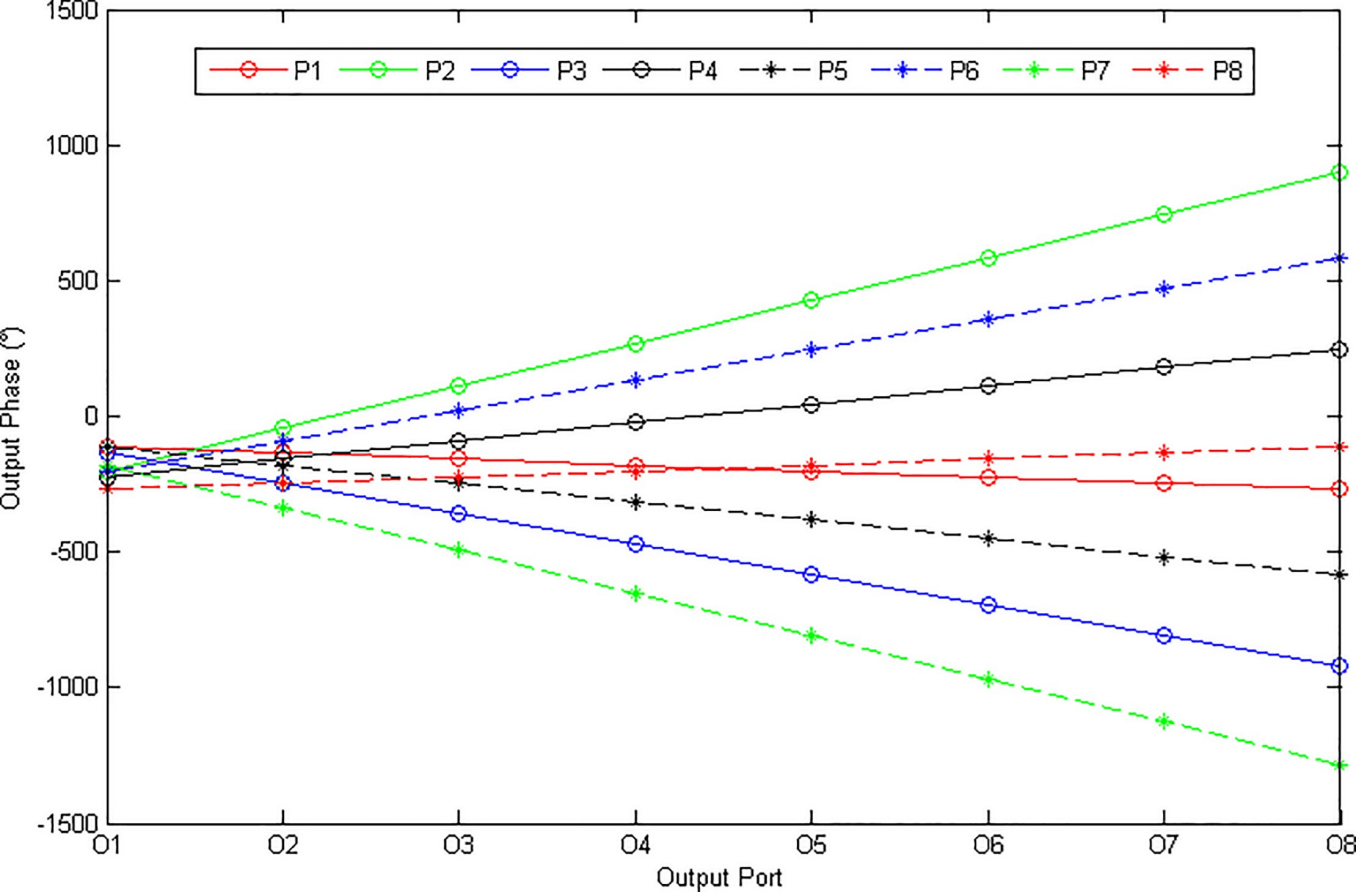
The ideal output phases of the 8 × 8 Butler matrix for the input ports.

The 8 × 8 Butler matrix consists of sixteen crossovers, twelve quadrature hybrids and eight phase shifters. The first value of the phase shifter is 90° – *ϕ*_*1*_ = 67.5°. The second value of the phase shifter is 90° – *ϕ*_*2*_ = 22.5° and the third value of the phase shifter is 90° – 2*ϕ*_*1*_ = 45°. Each circuit element of the Butler matrix was designed using a microstrip transmission line. The ideal output phases of an 8 × 8 Butler matrix are presented in Fig 2. As illustrated in the figure, the minimum phase differences were produced by ports P1 and P8. Meanwhile, the maximum phase differences were produced by ports P2 and P7.

## Circuit design using electromagnetic simulation software

### Simulation condition

The structure of the 8 × 8 Butler matrix was designed using electromagnetic (EM) simulation software called Computer Simulation Technology (CST) Microwave Studio. NPC-F220A from Nippon Pillar Packing Co. Ltd was used as the substrate in this work. The simulation parameters and the details of the substrate are summarized in Table 2.

**Table 2:** The simulation parameters and the details of the substrate.

Parameter	Detail/Value
EM simulator	CST Microwave Studio
Frequency	28 GHz
Type of substrate	NPC-F220A (Nippon Pillar Packing Co. Ltd.)
Dielectric constant	2.2
Tan *δ*	0.0007
Substrate thickness	0.254 mm

### Phase delay and loss of the microstrip transmission line

**Fig 3:**
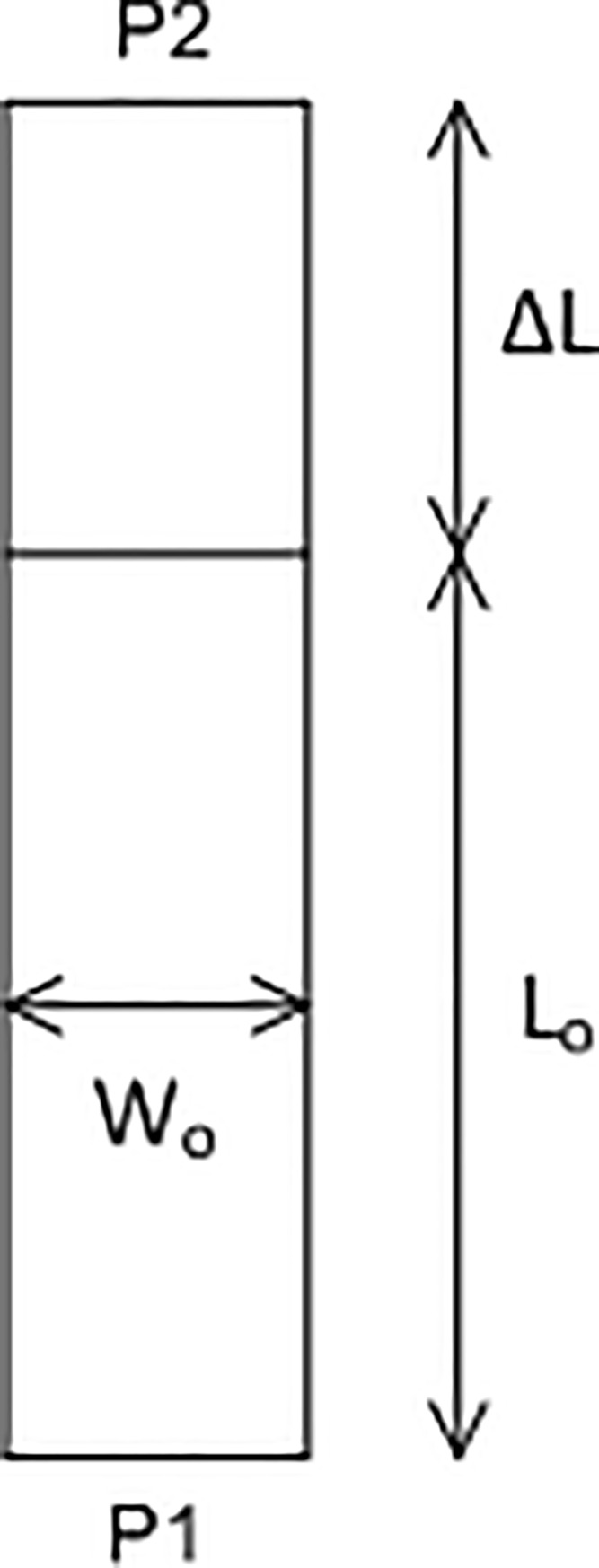
Model of the microstrip transmission line.

In the millimeter wave, it is crucial to ensure the dimensional accuracy of the circuit design. Therefore, the changes of phase in the microstrip transmission line were investigated to achieve a highly accurate circuit design. Fig 3 shows a model of the microstrip transmission line. The length and width of the microstrip transmission line are 7.7155 mm and 0.7826 mm, respectively.

**Fig 4:**
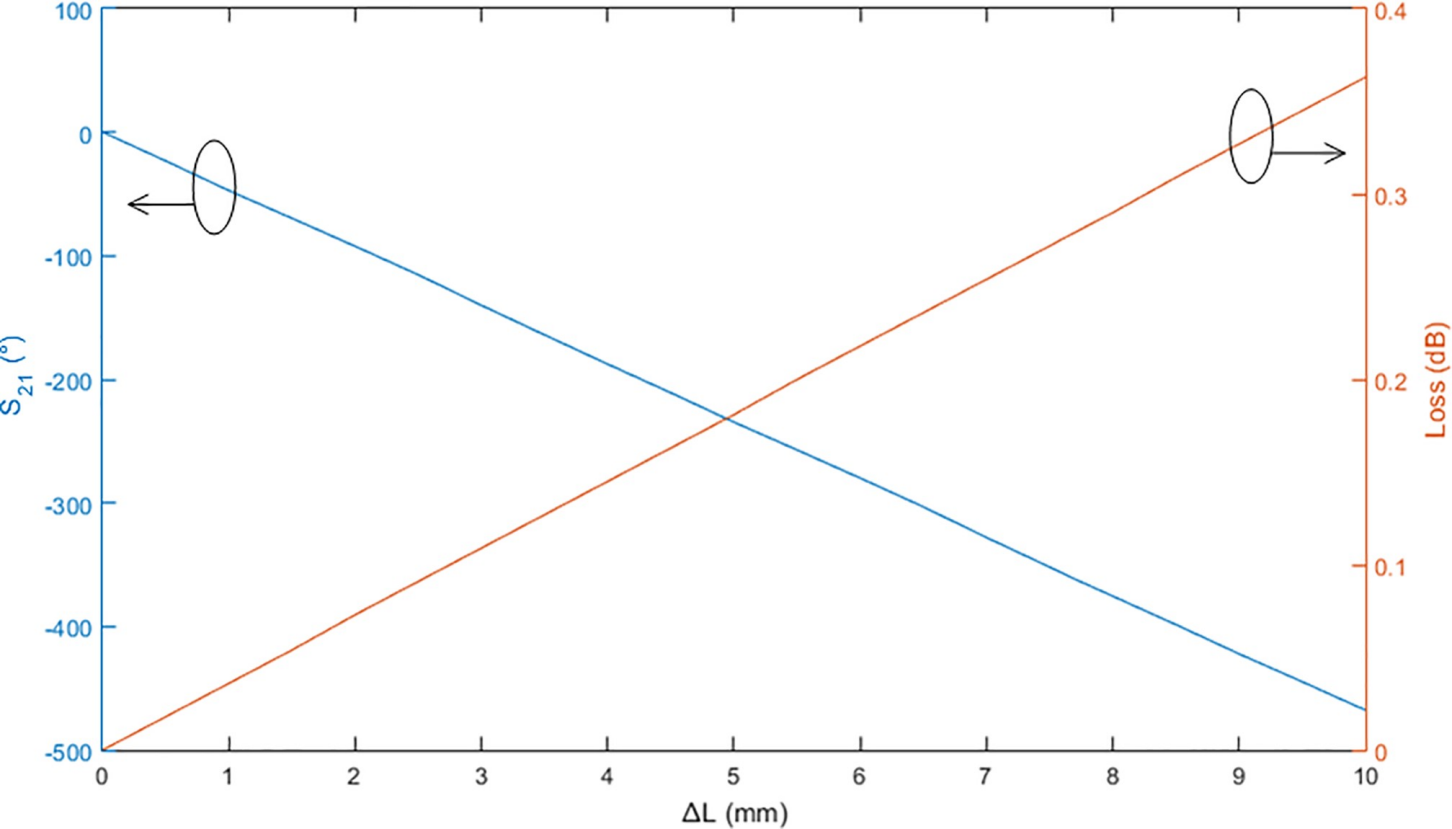
The phase of S_21_ and the line loss of the microstrip transmission line.

Based on the simulation, the phase of S_21_ of the extended lines is shown in Fig 4. The wavelength in the microstrip transmission line was *λ*_*g*_ = 7.663 mm, which corresponds to a 360° phase change. Therefore, a 1° phase change corresponds to a line length of 0.02 mm. To achieve an accurate output phase at 28 GHz, very precise dimensions of the circuit elements are required. Besides that, the loss of the microstrip transmission line is shown in Fig 4, approximately 0.363 dB for a line length of 10 mm.

### Crossover

**Fig 5:**
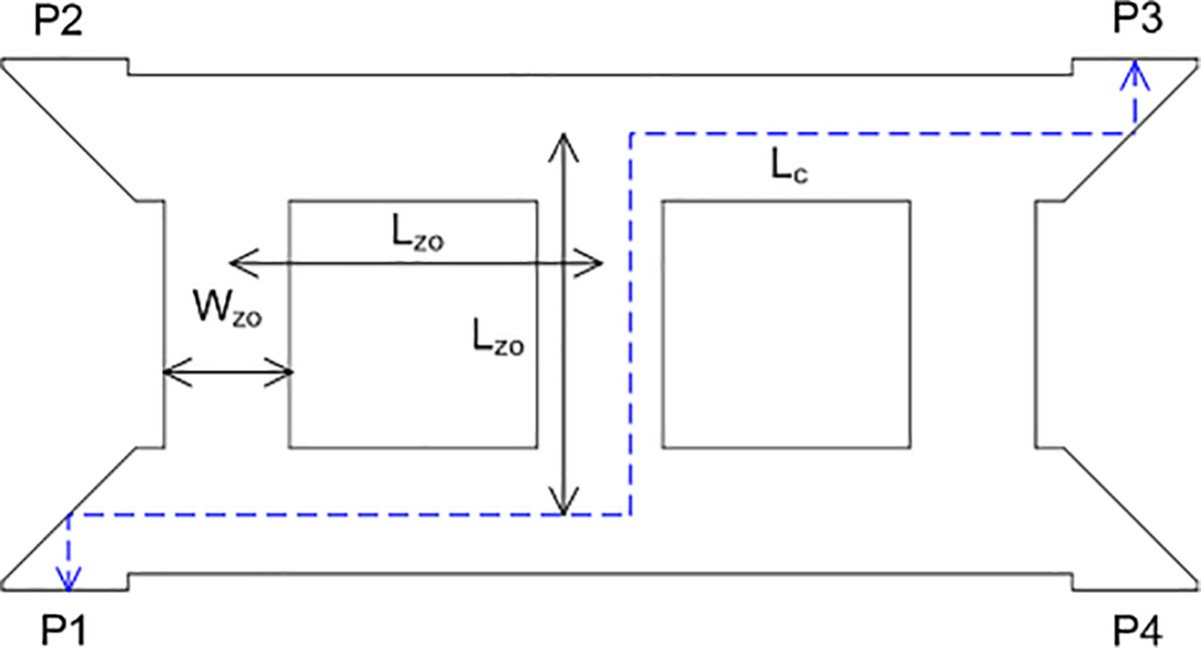
The geometry of the crossover.

The geometry of the crossover used in this study is illustrated in Fig 5. This circuit allows the signal to flow to cross over with high isolation. The signal flowed to P3 when P1 was excited. In contrast, the signal flowed to P2 when P4 was excited. Theoretically, the insertion loss of the crossover should be zero. However, it is practically impossible to achieve zero insertion loss. Therefore, a very accurate design is required to minimize insertion loss.

**Fig 6:**
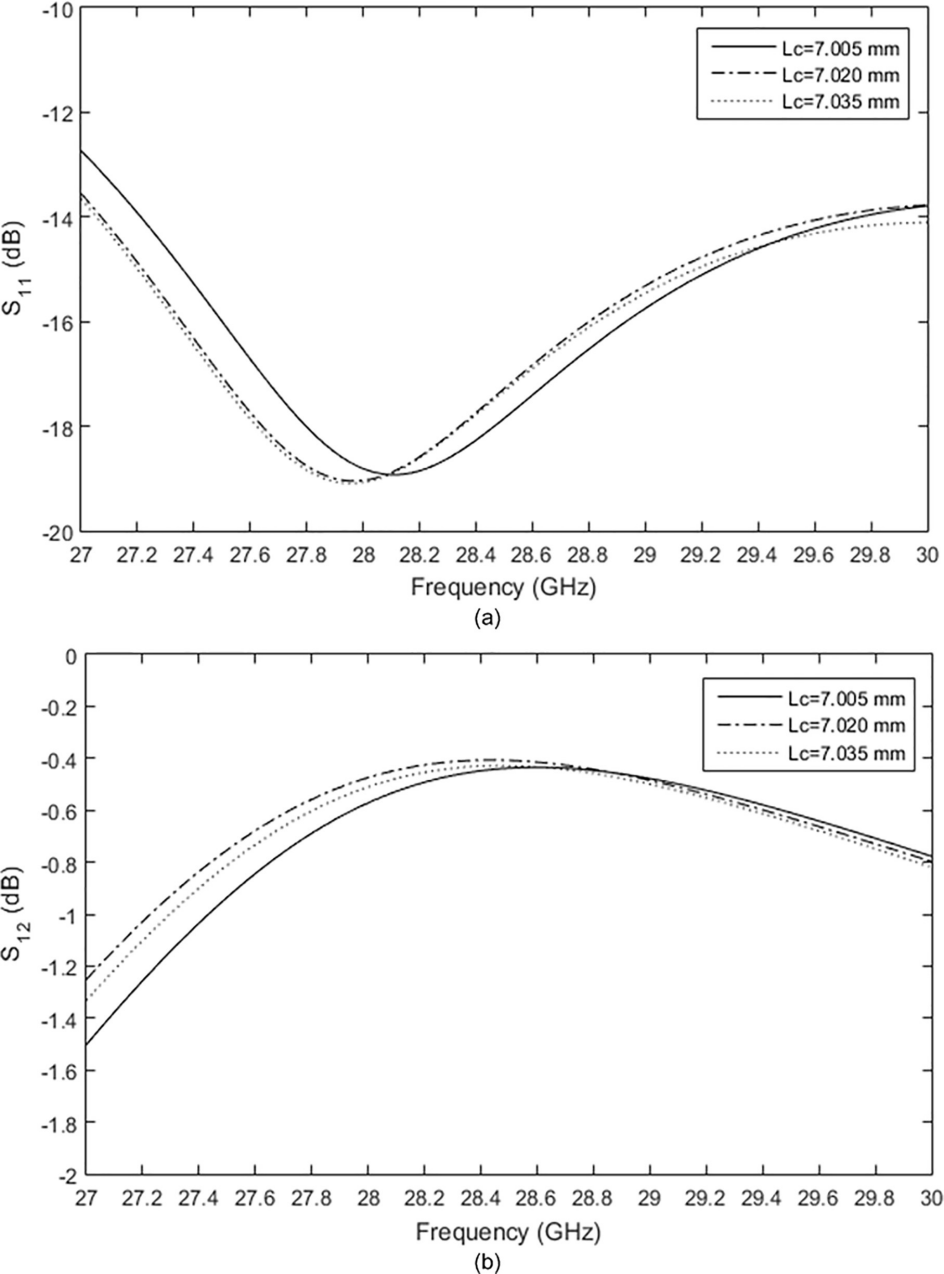
The effect of the total length of the crossover on (a) return loss and (b) insertion loss.

The crossover was designed using a 50 Ω microstrip transmission line. The width of *W*_*zo*_ was determined to achieve an impedance of 50 Ω and the length of *L*_*zo*_ was one-quarter wavelength. To design a crossover with a low insertion loss at 28 GHz, parametric studies were carried out. The most important parameter was the total length of the crossover from P1 to P3, which was labeled as *L*_*c*_, as shown in Fig 5. The length of *L*_*c*_ was varied and the optimum length was obtained via simulation. Fig 6 shows the effect of *L*_*c*_ on the return loss, S_11_ and the insertion loss, S_21_. As shown in these figures, the return loss and insertion loss were sensitive to slight changes in *L*_*c*_. For *L*_*c*_ = 7.005 mm and 7.020 mm, the return losses resonated at 28 GHz, but the return losses shifted to 28.2 GHz when *L*_*c*_ was increased to 7.035 mm. The lowest insertion loss of −0.4 was achieved when *L*_*c*_ = 7.020 mm.

**Fig 7:**
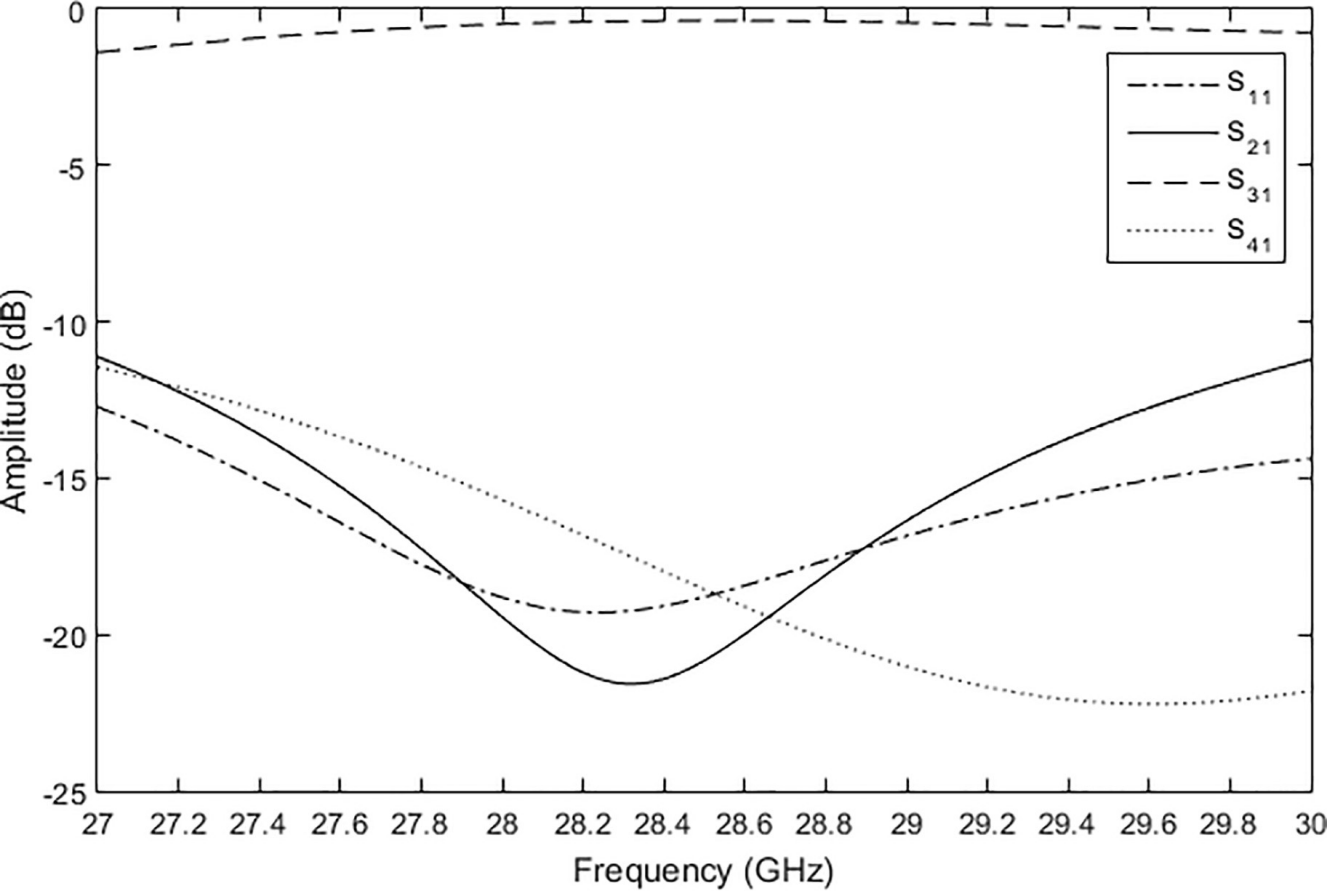
The simulated amplitudes of the crossover.

**Fig 8:**
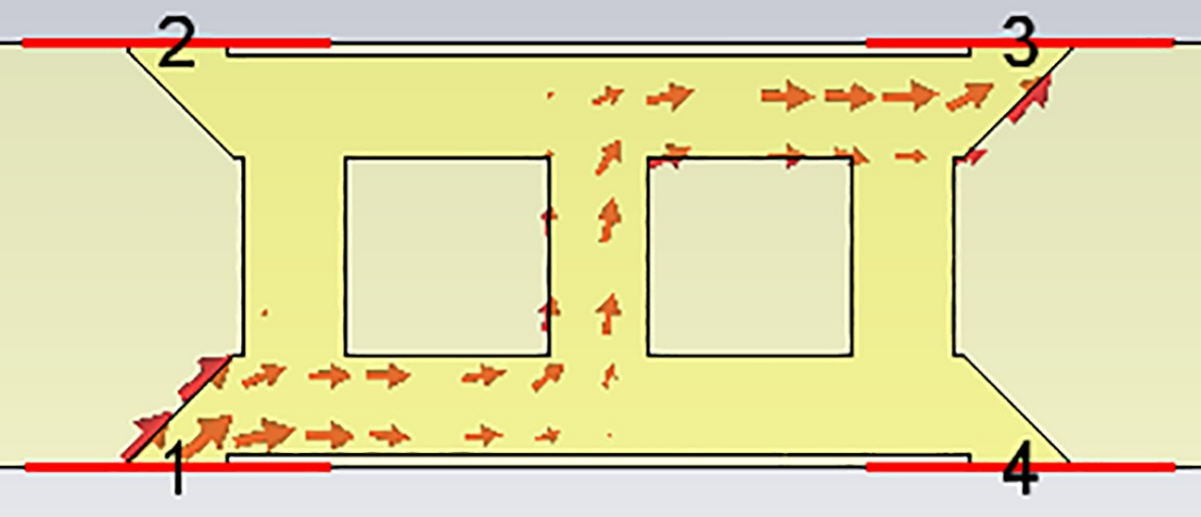
The power flow of the crossover.

Based on these parametric studies, the length of *L*_*c*_ = 7.020 mm was selected to simulate the crossover. The length, *L*_*zo*_ and width, *W*_*zo*_ of the crossover were 2.341 mm and 0.7826 mm, respectively. Fig 7 shows the amplitudes of each port of the crossover. The amplitude of the insertion loss, S_31_ was −0.4 dB at 28 GHz. This excellent result shows that the crossover had a very good design. The electric power flow of the crossover is shown in Fig 8.

### Quadrature hybrid

**Fig 9:**
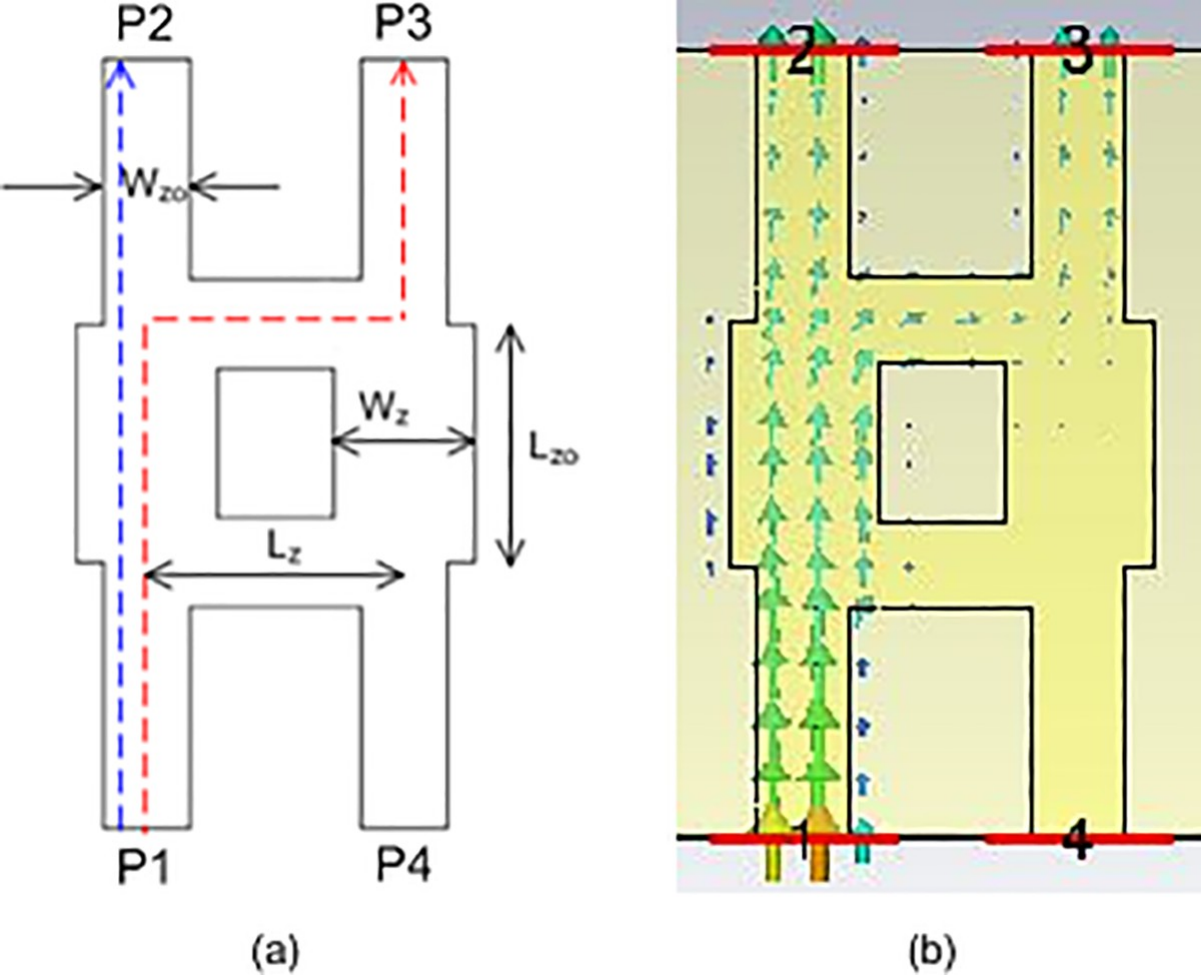
(a) The geometry and (b) the power flow of the quadrature hybrid.

The geometry of a quadrature hybrid is presented in Fig 9(A). The input power at port P1 was divided equally between output ports P2 and P3. The phase of port P3 was shifted by 90°. There was no power coupled to port P4, as the port was isolated. The important design parameters were lengths of *L*_*zo*_ and *L*_*z*_, which were required to achieve 90° at port P3. The widths of *W*_*zo*_ and *W*_*z*_ were determined to achieve impedances of 50 Ω and 35 Ω, respectively. The structure was simulated and the parameters of the quadrature hybrid are shown in Table 3. The length of *L*_*z*_ was almost one-quarter wavelength and the length of *L*_*zo*_ was slightly longer than *L*_*z*_ to achieve good isolation at port P2. The electric power flow of the quadrature hybrid is shown in Fig 9(B).

**Table 3:** The parameters of the quadrature hybrid.

Parameter	Value (mm)
*L*_*zo*_	2.341
*L*_*z*_	2.076
*W*_*zo*_	0.7826
*W*_*z*_	1.2754

**Fig 10:**
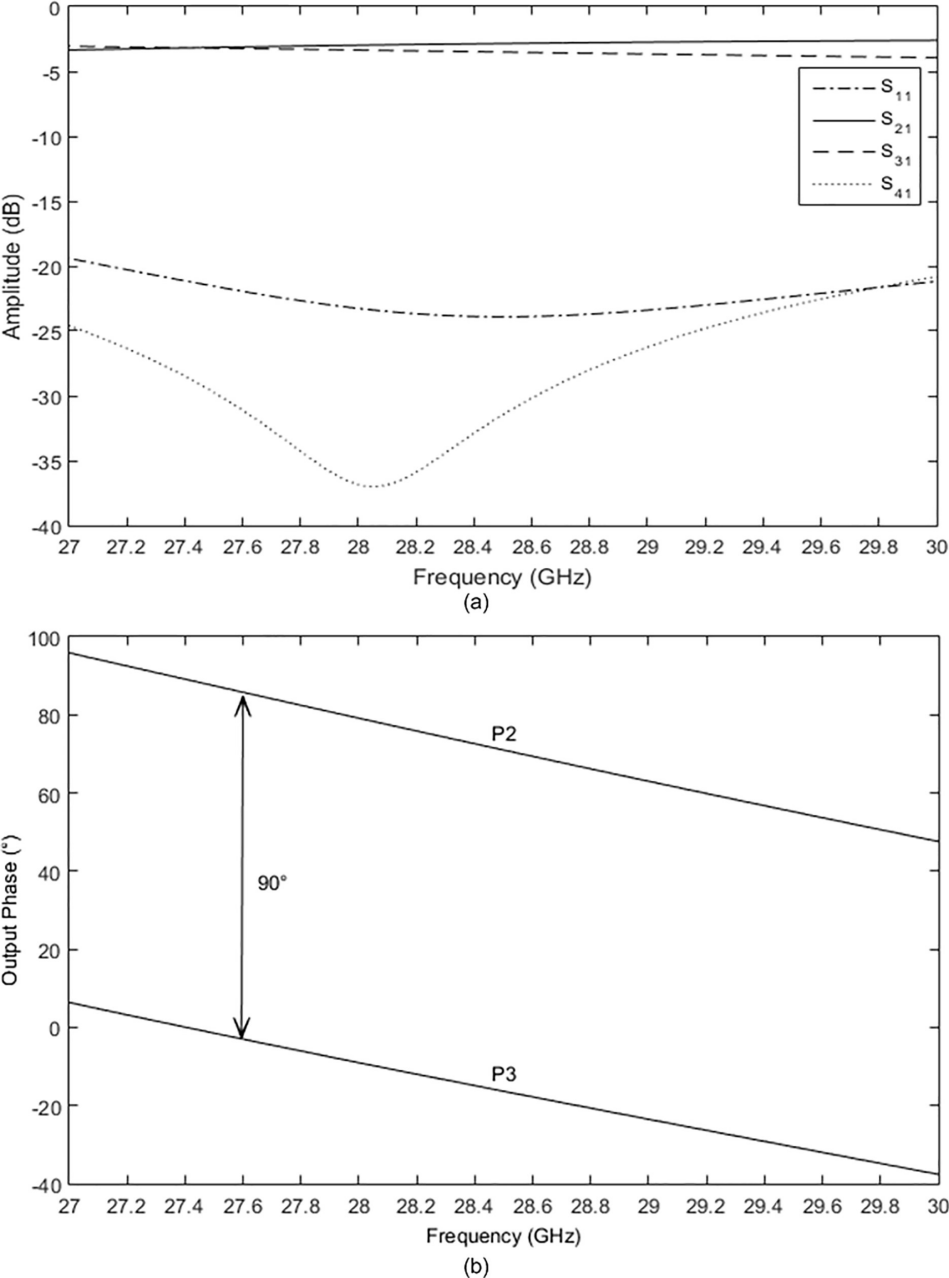
(a) The simulated amplitudes of the quadrature hybrid and (b) the output phases of ports P2 and P3 when P1 was excited.

The amplitudes of each port of the quadrature hybrid are shown in Fig 10(A). The amplitudes of S_21_ and S_31_ were approximately −3 dB at 28 GHz. Good isolations of S_11_ and S_41_ were achieved with less than −20 dB from 27 GHz to 30 GHz. Fig 10(B) illustrates the simulated output phases of ports P2 and P3 when P1 was excited. It can be observed that the phase difference between ports P2 and P3 was 90° in a wide frequency range of 27 GHz to 30 GHz.

### Phase shifters

**Fig 11:**
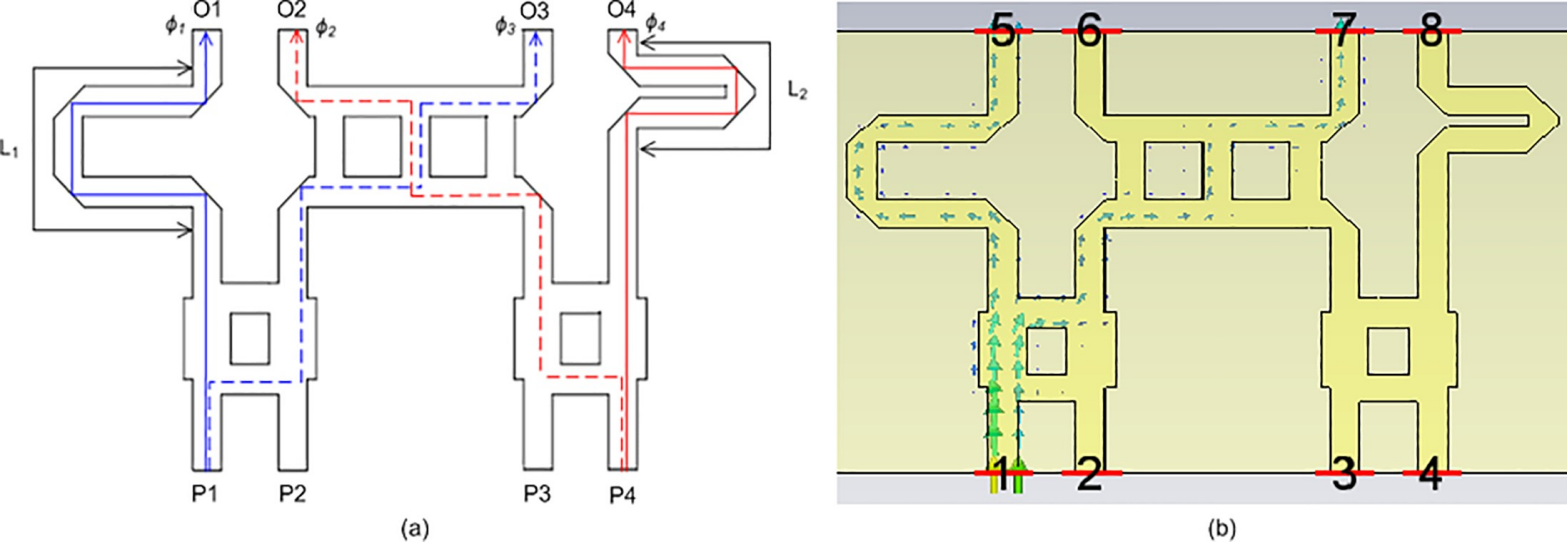
(a) The structure and (b) the power flow of the phase shifters in the Butler matrix.

**Fig 12:**
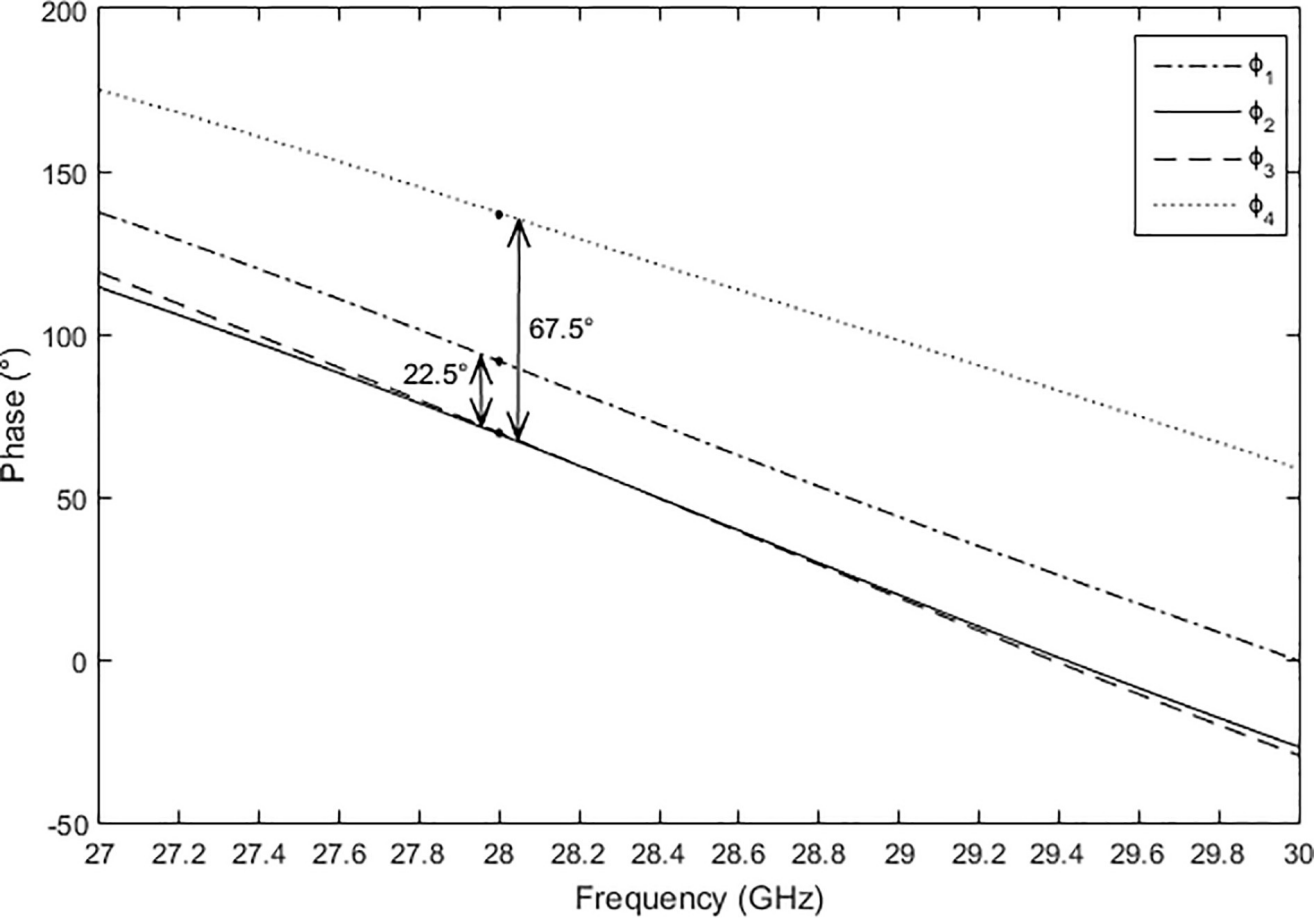
The phases of *ϕ*_*1*_ to *ϕ*_*4*_ at the output ports of the Butler matrix.

The phase shifters were designed using a 50 Ω microstrip transmission line. Fig 11(A) shows two phase shifters in the Butler matrix, labeled *L*_*1*_ and *L*_*2*_. The lengths of *L*_*1*_ and *L*_*2*_ were 11.9236 mm and 7.9652 mm, respectively. These different lengths were required to achieve phase differences of −67.5° and −22.5°. The phase differences can be expressed by Eq (2) and Eq (3). Fig 11(B) presents the electric power flows from port 1 to ports 5 and 7. Fig 12 illustrates the output phases of *ϕ*_*1*_ to *ϕ*_*4*_ at the output ports of the Butler matrix. The phase characteristics of *ϕ*_*2*_ and *ϕ*_*3*_ were the same due to the symmetrical structure.

$$\phi_{3}-\phi_{1}=-67.5{}^{\circ}$$ (2)

$$\phi_{2}-\phi_{4}=-22.5{}^{\circ}$$ (3)

### The 8 × 8 Butler Matrix

**Fig 13:**
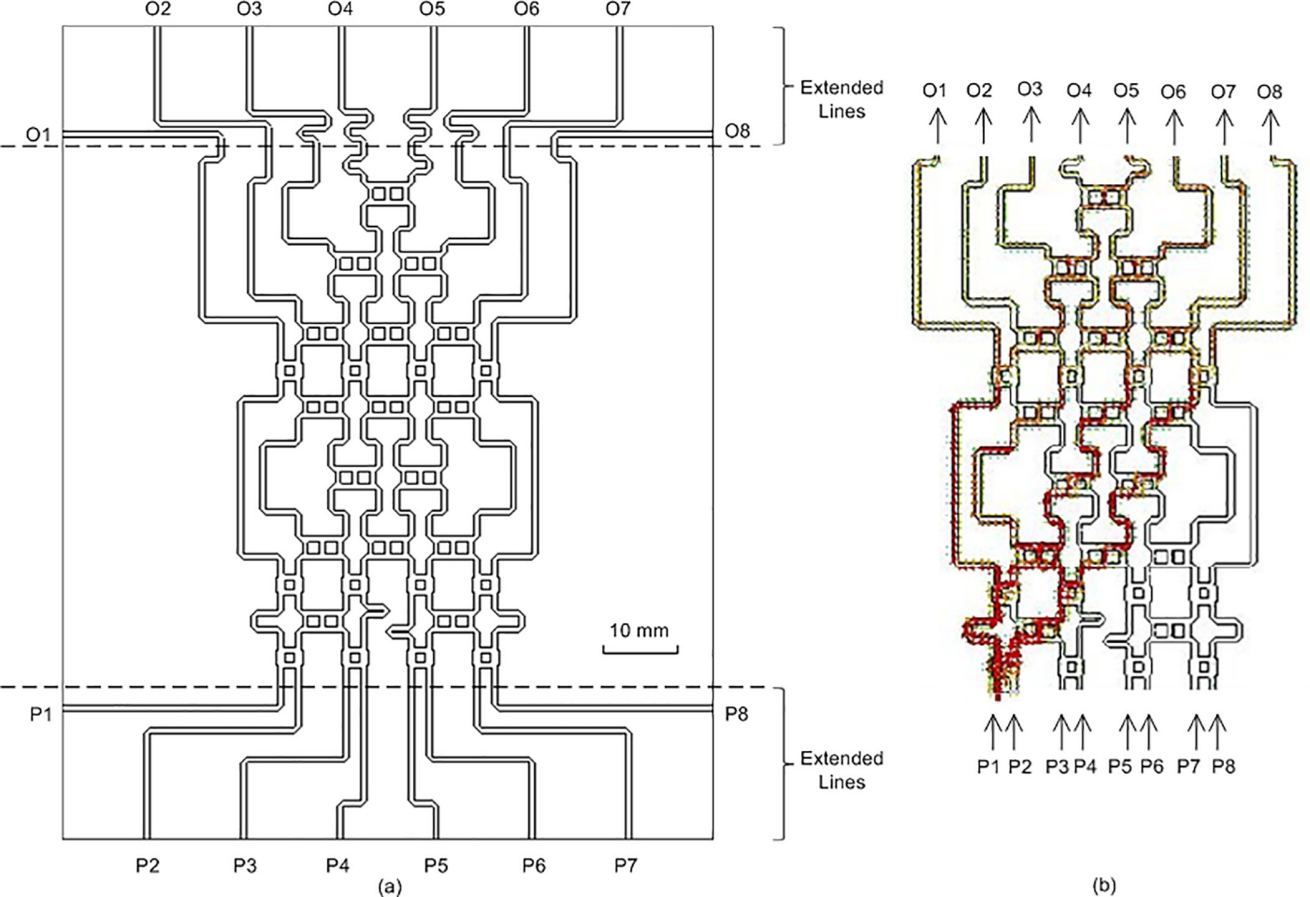
(a) The designed structure and (b) the power flow of the 8 × 8 Butler matrix.

Fig 13(A) shows the complete design structure of the 8 × 8 Butler matrix. As shown in this figure, the 8 × 8 Butler matrix has eight input ports (P1 to P8) and eight output ports (O1 to O8). Extended lines were designed to allow the implementation of coaxial connectors at the input and output ports of the 8 × 8 Butler matrix. Fig 13(B) shows the power flow of the 8 × 8 Butler matrix. As shown in this figure, the power supplied from port P1 flowed to the output ports, O1 to O8.

**Fig 14:**
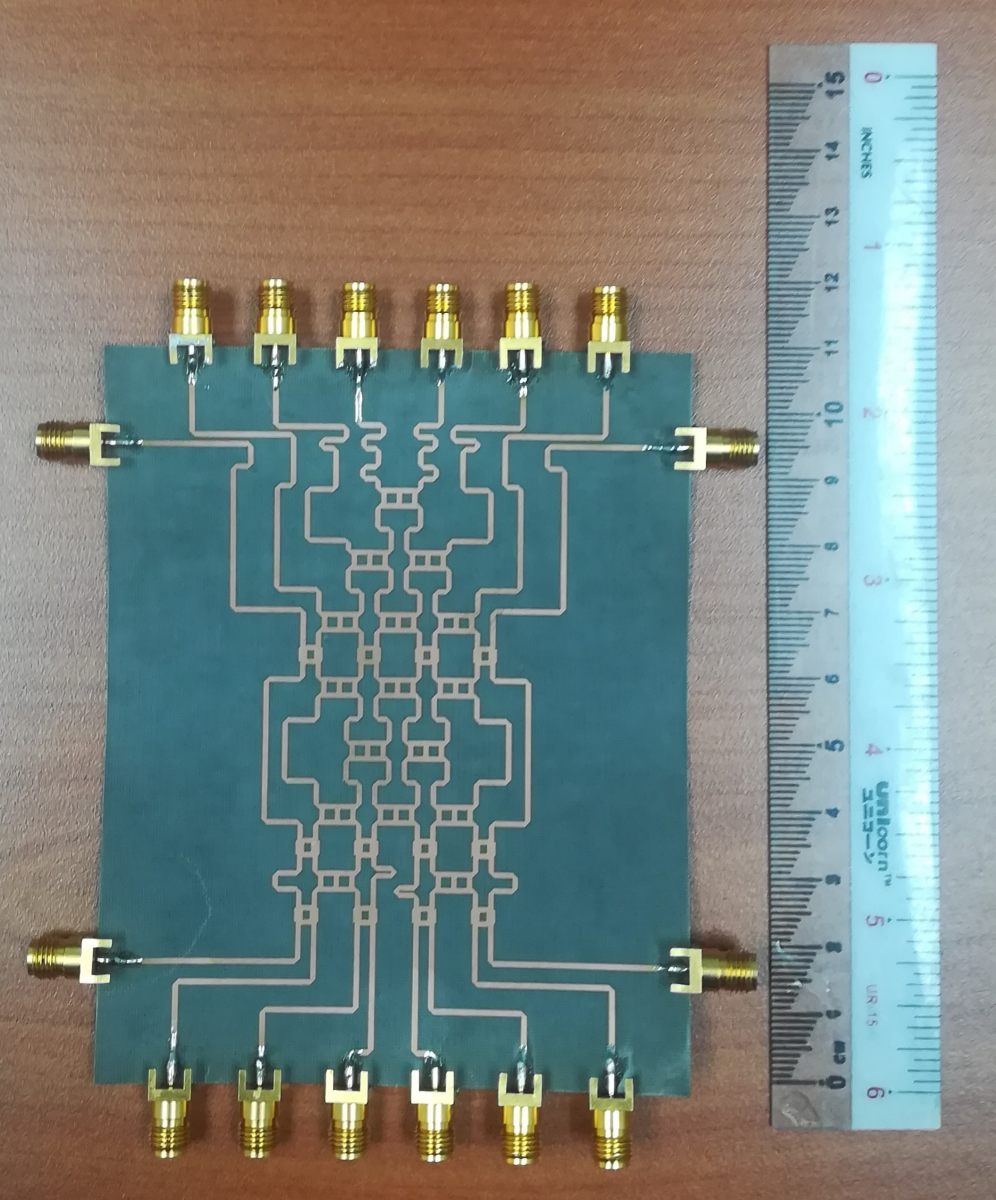
The photograph of the fabricated 8 × 8 Butler matrix.

The designed structure of the 8 × 8 Butler Matrix in Fig 13(A) was fabricated using NPC-F220A substrate via a photo etching process. Fig 14 shows a photograph of the fabricated 8 × 8 Butler matrix. The 2.92 mm coaxial connectors were used at the input and output ports of the 8 × 8 Butler matrix. The size of the fabricated board was 110 mm × 88 mm.

## Results and discussion

Two measurements were carried out in this work, namely return loss and transmission characteristics. The return loss measurement is a one-port measurement that measures the impedance matching of the input port. The transmission characteristics measurement is a two-port measurement to measure the amplitude and phase between the input and output ports of the Butler matrix. Both measurements were carried out using a vector network analyzer (Keysight N5224A).

**Fig 15:**
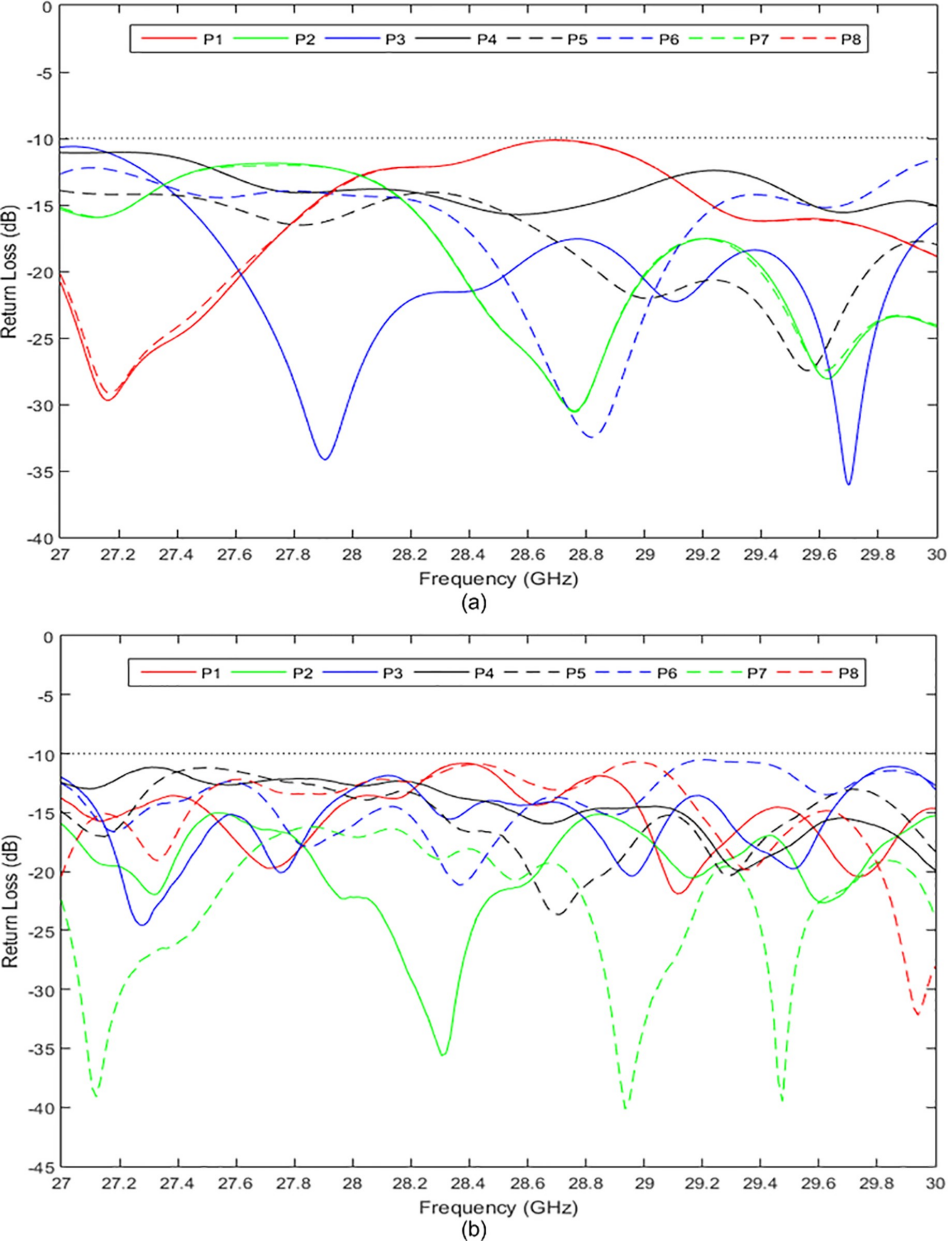
The (a) simulated and (b) measured return losses at the input ports of the 8 × 8 Butler matrix.

**Fig 16:**
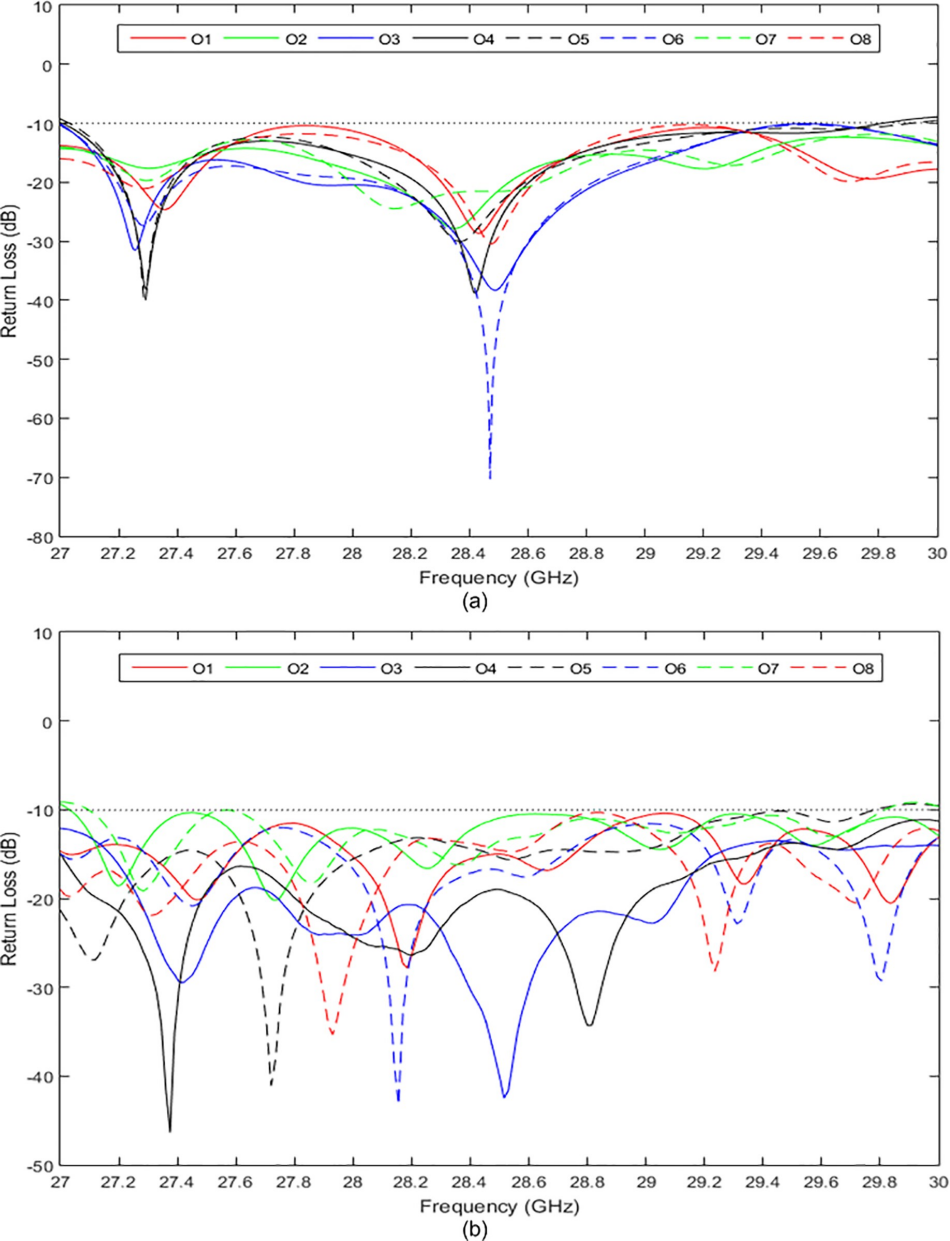
The (a) simulated and (b) measured return losses at the output ports of the 8 × 8 Butler matrix.

**Fig 17:**
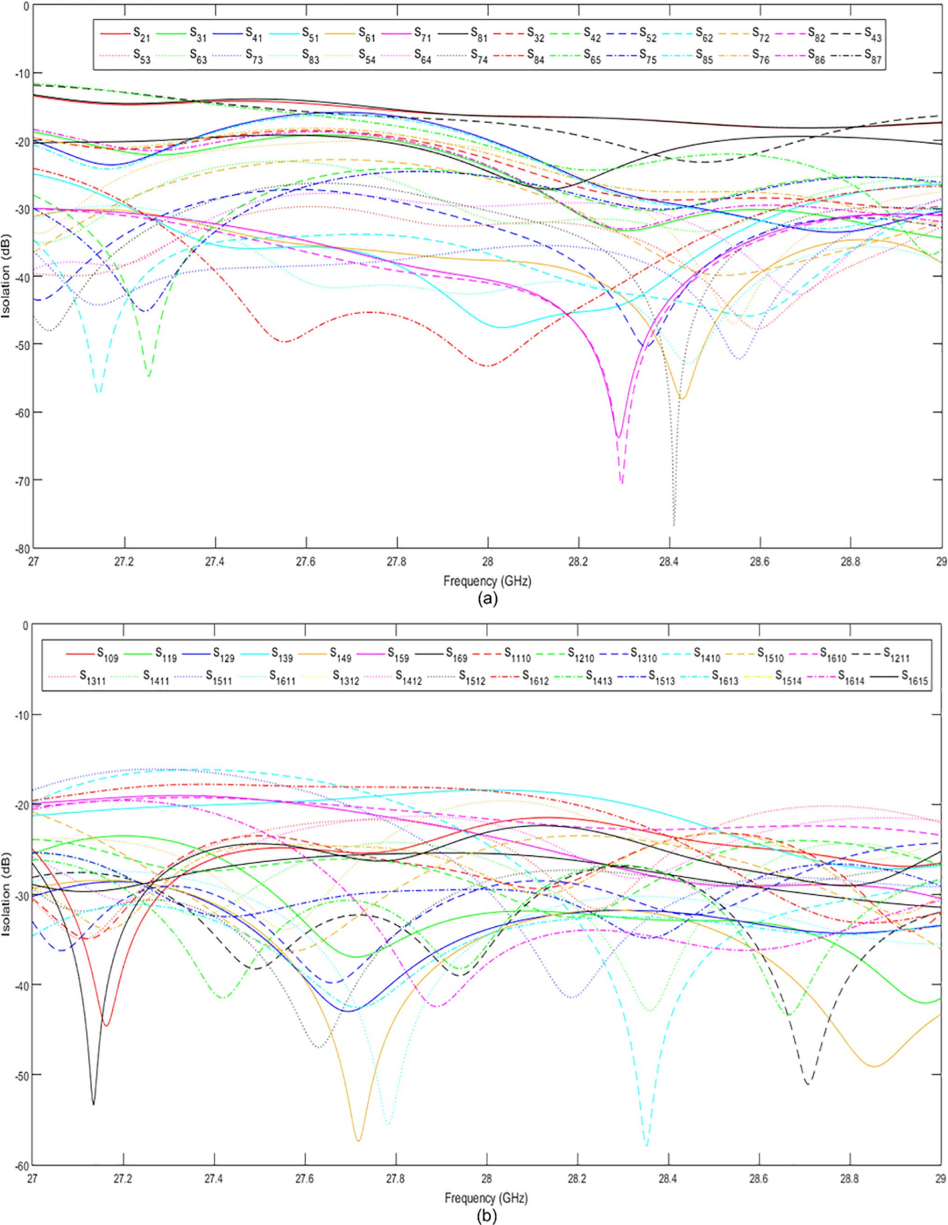
The simulated isolations between the (a) input and (b) output ports of the 8 × 8 Butler matrix.

**Fig 18:**
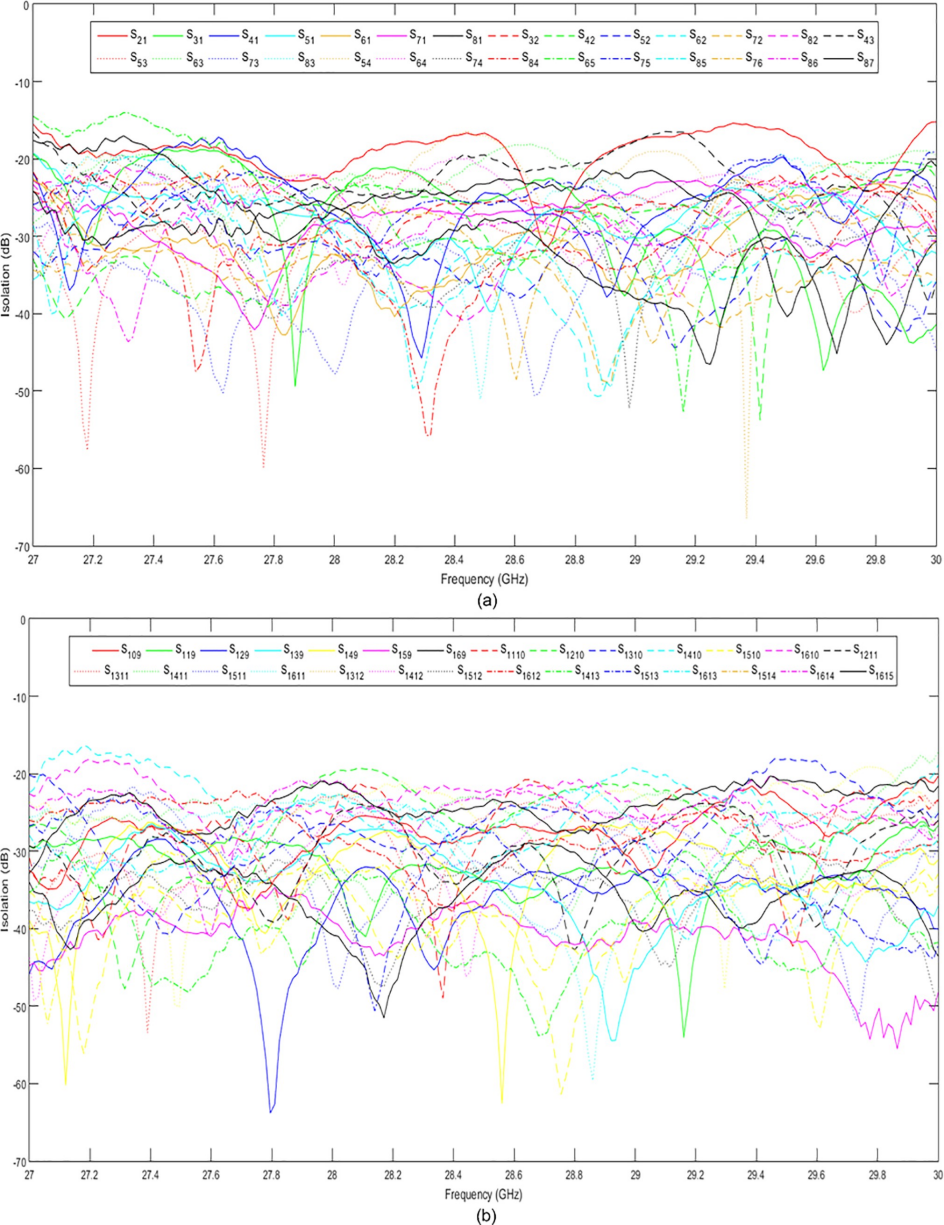
The measured isolations between the (a) input and (b) output ports of the 8 × 8 Butler matrix.

Fig 15(A) and 15(B) show the simulated and measured return losses at the input ports, respectively. Both the simulated and measured return losses were less than −10 dB in a wide frequency range of 27 GHz to 30 GHz. The simulated and measured return losses at the output ports are presented in Fig 16(A) and 16(B), respectively. The return losses were less than −10 dB between 27 GHz and 29.8 GHz for both simulation and measurement. It can be observed that the measured results were slightly different from the simulated results by 1.3%. This value is within an acceptable range due to the fabrication tolerance and effect caused by the coaxial connectors. The simulated and measured isolations between the input and output ports of the 8 × 8 Butler matrix are presented in Fig 17 and Fig 18, respectively. Both simulated and measured isolations were less than −15 dB at 28 GHz. The fabricated 8 × 8 Butler matrix was proven useful at the 28 GHz band.

**Fig 19:**
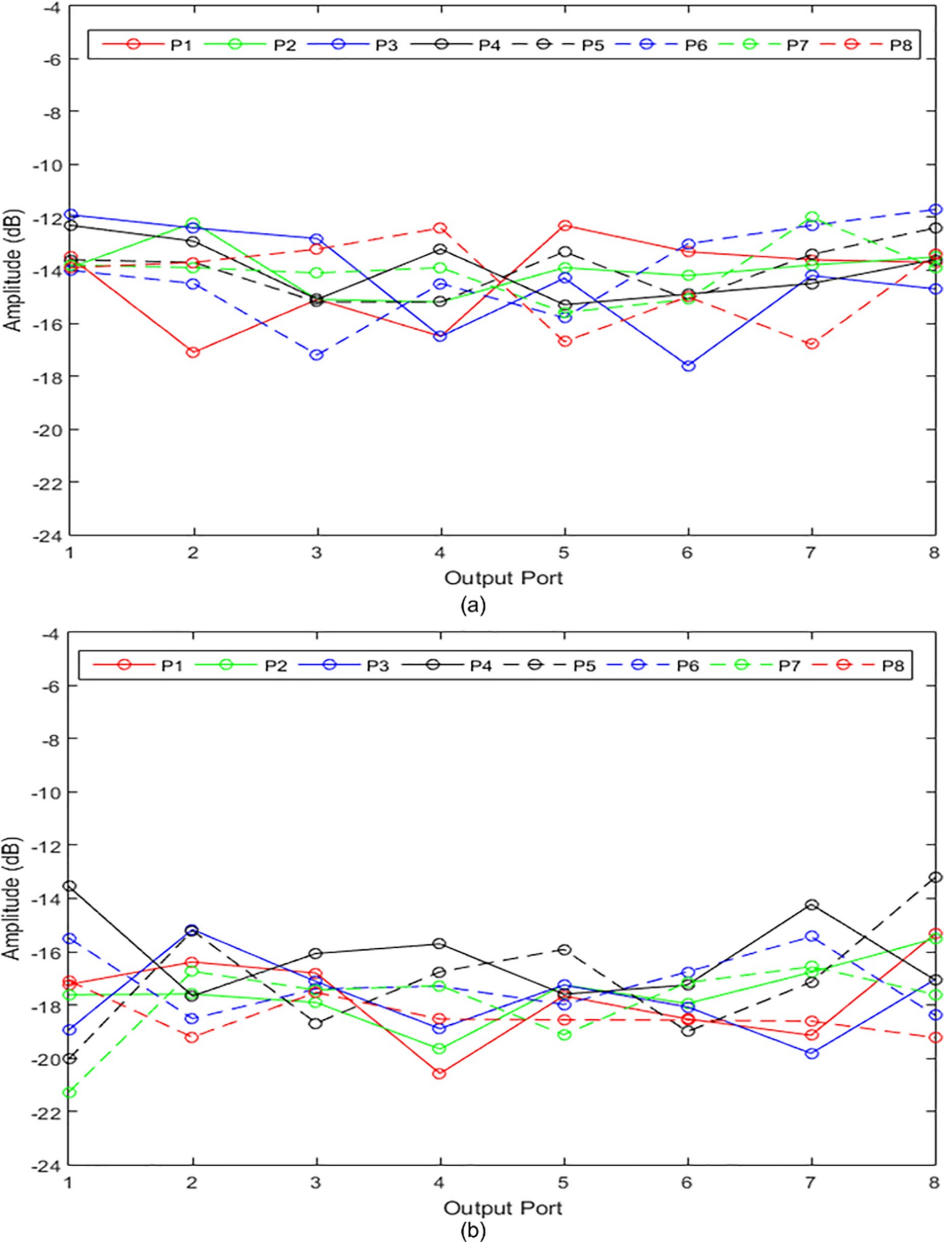
The (a) simulated and (b) measured transmission amplitudes at 28 GHz for the input ports of the 8 × 8 Butler matrix.

**Fig 20:**
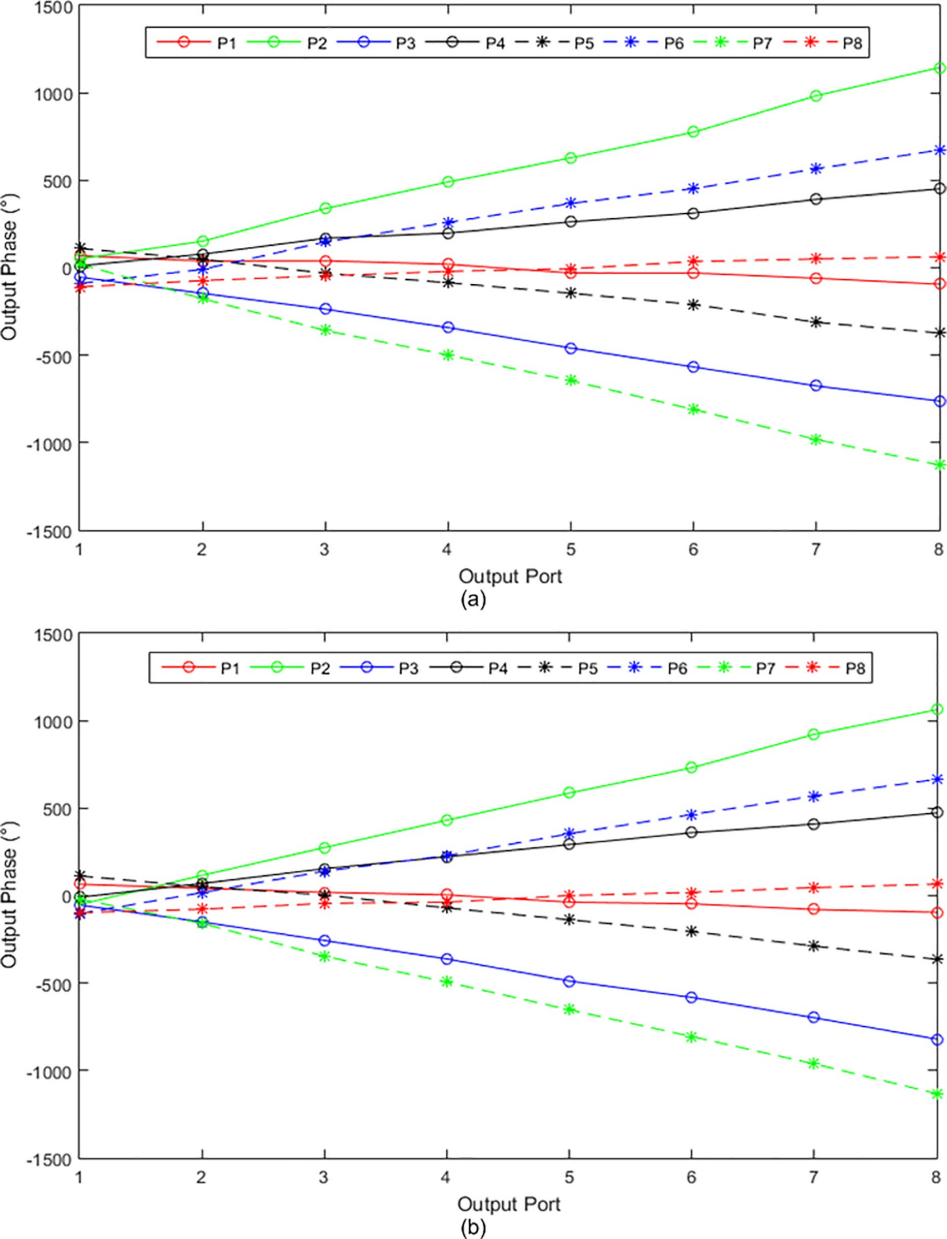
The (a) measured and (b) simulated output phases at 28 GHz for the input ports of the 8 × 8 Butler matrix.

Fig 19(A) and 19(B) show the simulated and measured transmission amplitudes of the output ports at 28 GHz, respectively. The transmission amplitude shows the ratio of the output power to the input power. Theoretically, the amplitude at each output port of the Butler matrix should be −9 dB when the input port is excited. However, the simulated amplitude was approximately −14 dB and the difference in the amplitude was −5 dB. Using the line loss of the microstrip transmission line in Fig 4, the loss was revealed to be 5 dB for a line length of 150 mm, which was estimated from the configuration in Fig 13(A). The average deviations of ± 2 dB were due to the reflections in the Butler matrix. For the measured transmission amplitudes, the average deviations of ± 2 dB were due to the fabrication tolerance and variations in the substrate properties. The average amplitudes were reduced from −15 dB to −17 dB. The measured output phases for the input ports at 28 GHz are illustrated in Fig 20(A). The measurement results in this figure were validated via comparison with the simulation results shown in Fig 20(B). A good agreement was obtained between the measured and simulated output phases with a phase error of ± 10°.

Table 4 summarizes the comparison between the proposed Butler matrix and previous works reported in the literature. Compared with the previous works, the insertion loss obtained in this study is almost the same. It is not difficult to achieve a low insertion loss at lower frequencies, as reported by Adamidis, Vardiambasis, Ioannidou and Kapetanakis [12] and Zhai, Fang, Ding and He [20] because the wavelength is larger. Zhong, Ban, Lian, Yang, Guo and Yu [21] reported a simulated insertion loss of ± 2 dB. In the study, the Butler matrix was designed based on a dual-layer structure in which the layers were connected via through-holes. However, the overall losses of the fabricated board were not clarified. The proposed Butler matrix in this study is a single-layer structure using printed circuit board technology, which is simple to design, cost-effective and easy to fabricate. Moreover, the proposed structure is compact and therefore the size of the Butler matrix is suited for a practical space-constrained base station compared to other antenna types such as reflector and lens antennas. In addition, it can be clearly observed that the proposed Butler matrix yielded the least phase error of ±10°.

**Table 4:** A comparison between the proposed 8 × 8 Butler matrix and previous works.

Reference	Technology	Number of layer	Frequency (GHz)	Circuit size (mm^2^)	Insertion Loss	Phase error
[12]	Printed circuit board	1	1.9	250 × 220	1.5 dB	± 12°
[20]	Printed circuit board	2	4.3	155 × 155	2.5 dB	± 15°
[21]	Substrate integrated waveguide	2	29.5	103 × 41	* 2 dB	* 15°
This work	Printed circuit board	1	28	110 × 88	± 2 dB	± 10°

*Simulation results

## Conclusion

In this paper, the design of a single-layer 8 × 8 Butler matrix operating at 28 GHz was proposed for a 5G base station antenna. The Butler matrix is compact with a dimension of 88 × 110 × 0.254 mm^3^. This circuit consisted of sixteen crossovers, twelve quadrature hybrids and eight phase shifters. These circuit elements were designed with highly accurate dimensions and the electrical performance was ensured via comprehensive simulation. In addition, the optimum design of the crossover, the quadrature hybrid and the phase shifter were demonstrated in detail. The 8 × 8 Butler matrix was fabricated using a low dielectric constant and a low loss tangent substrate material called NPC-F220A. The return losses were less than −10 dB in a wide frequency range of 27 GHz to 30 GHz. The insertion loss and phase error were significantly improved, yielding values of ± 2 dB and ± 10°, respectively. A good agreement between the measured and simulated results was obtained in this work. Therefore, the proposed 8 × 8 Butler matrix can be used for a 5G base station antenna.
